# Farmers' practices and their knowledge of biotic constraints to sweetpotato production in East Africa^☆^

**DOI:** 10.1016/j.pmpp.2018.07.004

**Published:** 2019-01

**Authors:** Richard Echodu, Hilary Edema, Godfrey Wokorach, Christine Zawedde, Geoffrey Otim, Nessie Luambano, Elijah Miinda Ateka, Theodore Asiimwe

**Affiliations:** a Faculty of Science, Gulu University, P.O. Box 166, Gulu, Uganda; b Gulu University Bioscience Research Laboratories, P.O. Box 166, Gulu, Uganda; c Faculty of Agriculture and Environment, Gulu University, P.O. Box 166, Gulu, Uganda; d Sugarcane Research Institute, P.O. Box 30031, Kibaha, Tanzania; e Department of Horticulture, Jomo Kenyatta University of Agriculture and Technology, P.O. Box 62000-00200, Nairobi, Kenya; f Rwanda Agriculture Board, P.O. Box 5016, Kigali, *Rwanda*

**Keywords:** Ipomoea batatas, Food security, Sweetpotato, Kenya, Uganda, Tanzania, Rwanda

## Abstract

Sweetpotato (*Ipomoea batatas*) is a vital crop for overcoming food insecurity in sub-Saharan Africa and its production is highest in East Africa where yields are high and the growing seasons are short. This cross-country study assessed farmers' local practices and their knowledge of the biotic constraints to sweetpotato production in Uganda, Rwanda, Kenya and Tanzania with the aim of providing empirical data that can ultimately be used to enhance sweetpotato production in these four countries. We collected data from 675 households using a standardized questionnaire integrated with a web-based mobile app. Survey results provided strong evidence that sweetpotato is valued as an important subsistence crop among smallholder farmers on pieces of land of less than 0.4 ha, and we observed that females were more involved than males in sweetpotato production. Sweetpotato was ranked as the second most important staple crop after cassava. Farmers noted an increase in sweetpotato production over the past five years in Uganda and Kenya but a decrease in Rwanda and Tanzania; the proportion of farmers who reported a decrease (33%) and an increase (36%) did not significantly differ. The main constraints to production were reported to be pests (32.6%), drought (21.6%), diseases (11.9%) and lack of disease-free planting materials (6.8%). Farmers recognized the signs and symptoms associated with sweetpotato diseases on leaves, root tubers, and whole plants, but most were unable to assign the disease type (bacterial, fungal or viral) correctly. We suggest that regional governments improve education, increase the provision of clean planting materials and strengthen breeding programs to improve disease resistance.

## Highlights

• Sweetpotato was ranked the second most important root crop after cassava.
• Sweetpotato is a subsistence crop grown by smallholder farmers on pieces of land of less than 0.4 ha.
• Cultivation is performed mostly by women.
• Pests (32.6%), drought (21.6%), diseases (11.9%) and lack of clean planting materials (6.8%) were the main production constraints.
• Plant diseases were the third most important sweetpotato production constraint after pests and drought in all four countries.
• We suggest that regional governments strengthen clean seed-vine multiplication and sweetpotato breeding programs for disease resistance.

## 1 Introduction

Sweetpotato (*Ipomoea batatas* (L.) Lam) is the seventh most important food crop in the world after rice, wheat, potato, maize, cassava and barley [1]. More than 107 million tons are produced globally each year—96.3% of which are in developing countries [2,3], specifically in Asia (82.3%) and Africa (14%) [4]. According to the United Nations Conference on Trade and Development, China is the world's largest sweetpotato producer, with 76.2% of global production [4]. Smallholder farmers in Africa value sweetpotato because it grows in a variety of climates with few inputs and can withstand drought [5]. Sweetpotato is rich in carbohydrates and vitamins A, B and C, as well as minerals like phosphorus, iron and calcium. It is also a good food-security crop and can be sold for cash by African women and poor families [[5], [6], [7], [8]].

East Africa is the major sweetpotato production region in Africa [9,10], however there are several constraints on its production there. Smallholder farmers lose 20–98% of their sweetpotato yields due to a range of factors including attack by around 300 species of arthropods [18] as well as at least 30 diseases [[19], [20], [21]]. Most notably, sweetpotato diseases have a large impact on food security and income generation in the region [11]. For instance, viral diseases alone can lead to sweetpotato yield reductions of up to 98% [12]. Other constraints to sweetpotato production include poor access to quality seed-vines of suitable varieties, drought/weather and poor agronomic management practices by farmers that in turn affect yield, food availability and income for households [22,23]. There is also limited land area for cultivation as the human population increases, and this leads to proliferation of pests and diseases [24]. Despite these challenges, there is a need to increase productivity and tackle its constraints. Improving sweetpotato production requires supply of and access to good quality seed-vines, accompanied by improvements in plant nutrition and disease and post-harvest management.

Sweetpotato remains a vital crop in overcoming food insecurity for the fast-growing population in sub-Saharan Africa because of its high yield in short growing seasons of rain-fed systems [13]. Annual sweetpotato production in Uganda, Kenya, Rwanda and Tanzania is 1.7 million, 1 million, 900,000 and 340,000 tons, respectively [16]. Annual consumption of sweetpotato is estimated at 73 and 24 kg/person in Rwanda and Kenya, respectively [4,17]. In Uganda, it is the third most-consumed staple food after banana and cassava, with annual consumption of 73 kg/person. In Rwanda, where sweetpotato was introduced via Uganda, the crop also played a major role in helping refugees maintain food security during the genocide period [14,15].

In Africa, subsistence farmers usually grow sweetpotato in small plots of less than 0.5 ha without inputs such as fertilizer. Sweetpotato is currently being developed to address vitamin A deficiency [25], one of the most serious health and nutrition problems of sub-Saharan Africa [26,27]. At the household level, women generally play the dominant role in production and utilization of sweetpotato; for example, in Uganda, women are most involved in the cultivation activities (planting, weeding and harvesting) and food preparation (peeling, slicing, drying and cooking of the lateral roots, referred to as root tubers). Men are typically more involved in the transportation and sales transactions of marketed sweetpotato [28].

The regional integration of the East African communities in recent decades has enhanced markets and productivity in Rwanda, Uganda, Kenya and Tanzania, resulting in increased population movement and trade between the countries. The extent of cross-border spread of sweetpotato pests and diseases remains unknown. Thus, there is a need to review sweetpotato production and ascertain farmers' local knowledge and practices in disease management. In addition, there have been limited regional studies to assess local management practices concerning sweetpotato diseases because individual countries have conducted most previous studies. Given the free movement of goods among Rwanda, Uganda, Kenya and Tanzania, concerted disease control efforts within one country will not be effective if there are many opportunities for reinvasion and spread from neighboring countries. This calls for a joint action plan from national agricultural research systems among the four countries for identifying, monitoring and controlling these diseases across these countries.

In this study, we undertook cross-sectional surveys in Uganda, Kenya, Rwanda and Tanzania with the goal of understanding local practices and farmers' knowledge on biotic constraints. We also assess the contribution of women in sweetpotato production in the four surveyed countries. Field observations of the symptoms of diseases on sweetpotato plants in each district complemented the surveys. This work aims to guide future strategies in sweetpotato production and food security in the region.

## 2 Materials and methods

### 2.1 Study area

**Fig. 1:**
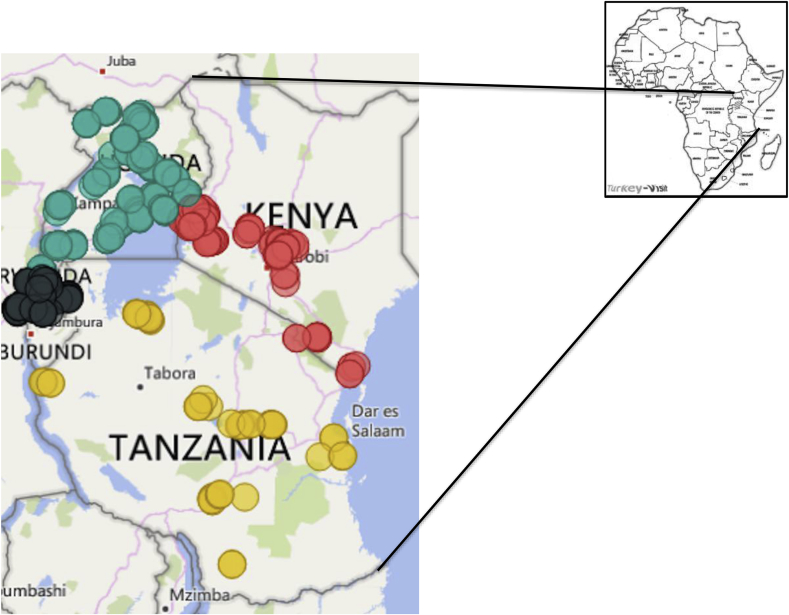
Map of Rwanda, Uganda, Kenya and Tanzania showing the localities surveyed in this study. Colored circles represent sampling sites in Uganda (363 sites, green), Kenya (110, red), Rwanda (101, black) and Tanzania (101, yellow). The inset map shows the geographical positions of the four countries in Africa. (For interpretation of the references to colour in this figure legend, the reader is referred to the Web version of this article.)

This study was conducted in the major sweetpotato-growing areas of Uganda, Kenya, Rwanda and Tanzania as identified by local experts within these countries. For logistical reasons, surveys were carried out in 2015, 2016 and 2017 in Uganda, in 2015 In Rwanda, and in 2016 in Kenya and Tanzania. Farm surveys in Uganda were done in Northern, Eastern, Western, Central and Northwest regions of the country, covering 25 districts (Fig. 1, Appendix 1). Of the four countries studied here, Uganda has the widest and most evenly distributed production of sweetpotato by geographical area, with most production occurring in the south close to Lake Victoria [29]. Uganda lies within the Nile Basin, and has mostly a plateau topology and a tropical rainy climate with two dry seasons: December–February and June–August.

In Kenya, surveys were conducted in the Western, Rift Valley, Nyanza, Eastern, Central and Coastal agroecological zones (AEZ), covering 14 districts. Kenya lies on the equator and overlies the East African Rift reaching up to Lake Victoria and southeast to the Indian Ocean. Climate varies, with cool and hot areas and coastal areas with tropical conditions.

In Rwanda, surveys were carried out in Southern, Western, Northern and Eastern AEZ, covering 12 administrative districts. Rwanda is a highly elevated country in the African Great Lakes region with mountains in the west and savanna in the east. The climate in Rwanda is temperate to subtropical, with two rainy seasons and two dry seasons each year.

Farm surveys in Tanzania were carried out in the Central, Eastern Coastal, Lake Victoria Zone, Southern Highlands and Western AEZ in 12 districts. Among these zones, the Lake Victoria, Southern Highlands and Eastern Coastal AEZ are among the highest sweetpotato-producing areas in Tanzania [30]. Tanzania is located within the African Great Lake Regions and has some parts in Southern Africa. The coastal climate is tropical with hot and humid conditions, and the northwestern highlands have cool and temperate conditions. The country has two rainy seasons: October–December and March–June. The central plateaus alternate between dry and humid throughout the year. The Lake Victoria AEZ is unique in that it includes all four countries, with much cross-border movement among farmers.

### 2.2 Farm selection, field observations and questionnaire administration

District agricultural officers and extension workers were used to identify villages with high sweetpotato production and about 10 households in each district were selected randomly, for a total of 675 households: 363 in Uganda, 110 in Kenya, 101 in Rwanda and 101 in Tanzania. Sweetpotato gardens were sampled by a systematic random-sampling strategy. Any garden that fell within 2 km along the road or established path in each of the villages was selected, as previously described [7,31,32]. Each of the gardens selected was at least 2-months-old to ensure the sweetpotato plants had developed mature leaves, which improves identification of disease symptoms. Each garden was divided by two transect lines as described by Mukasa et al. [12]. Field observations were done along the transect lines to identify vines showing symptoms of sweetpotato viral infections which included: leaf curl, leaf mosaic, vein clearing, mottling, leaf distortion, yellow chlorosis, purple chlorosis, necrotic spots, and stunting [[33], [34], [35]]. Farmers' knowledge of disease states were tested using the actual plants in their fields and also given images of symptoms.

A structured standard questionnaire issued upon visits to farmers was used to collect the following information: ranking of sweetpotato as a staple in the household, the family's total cultivated land area, total area under sweetpotato, sweetpotato production trends, constraints to sweetpotato production they have experienced or observed, local cropping practices, seedbed types, sources of planting materials, knowledge of sweetpotato disease symptoms and insect pests, and biotic constraints. The GPS coordinates of the field were also recorded on the questionnaire by the research team.

The questionnaire was pre-tested in each country before being administered, and revisions were made. The final questionnaire was developed into a mobile survey app using the ZERION iFormBuilder online service (Zerion Software Inc., Herndon, VA, USA) and then installed onto smart phones and tablets. These devices were carried in the field with portable solar power backups. All data collection in Uganda and Rwanda was done prior to development of the app, so the paper-based survey data were transferred into the app later and validated.

Ethical standards were maintained throughout the surveys. Country team leaders, local community leaders and agricultural extension staff or district agricultural officers, as mentioned earlier, were involved in the farm surveys. The farmers were briefed on the goal of the research and consent was obtained from each farmer interviewed.

### 2.3 Data collection

The surveys provided multiple-choice answers along with the ability to add a text response to a question if the existing list of predefined answers did not cover a response from a farmer. Care was taken to validate each data entry; for example, using the app software, longitude and latitude entries were confined to minimum and maximum values for a given country.

### 2.4 Data analysis

**Fig. 2:**
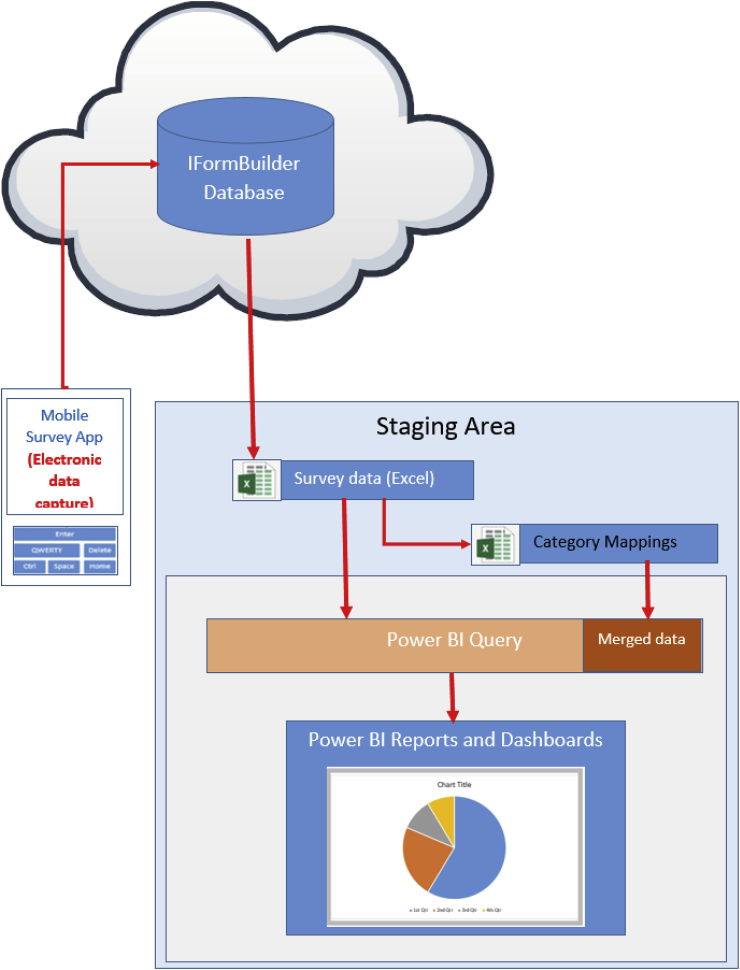
Conceptual model from data capture to analysis.

Data were exported from the iFormBuilder database and then loaded into the analytics software Power BI (Microsoft, Redmond, WA, USA) using the Power Query tools available (Fig. 2). Any manual text entries were cleaned and mapped to a common format or category. The listing and mapping of data were done using Excel, and then the mapped list was imported and merged with the relevant Power BI dataset.

Z-tests were used to compare the proportions of female to male participation in sweetpotato production in households. Z-tests were also used to compare the proportions and differences among countries in the land used to cultivate sweetpotato and other crops.

## 3 Results

### 3.1 Determination of local practices in sweetpotato production in Uganda, Kenya, Rwanda and Tanzania

Our assessment of local practices included (1) the role of farmer gender in sweetpotato production and the ranking of sweetpotato as a staple crop, (2) sweetpotato production trends over a five-year period, (3) sweetpotato cultivated area, (4) observed constraints in sweetpotato production, (5) seedbed type and (6) the sources of farmers' planting material.

#### 3.1.1 Farmer participation in sweetpotato production by gender

**Fig. 3:**
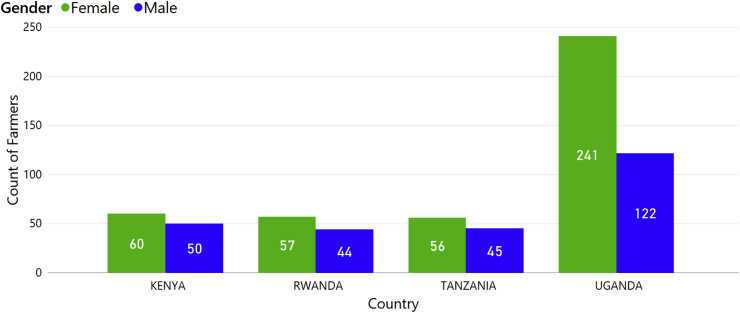
Numbers of farmers surveyed by gender and country: Uganda (n = 363 surveys), Kenya (n = 110), Tanzania (n = 101) and Rwanda (n = 101).

To assess local practices in sweetpotato production and farmers' knowledge on sweetpotato diseases, we carried out a total of 675 farm surveys in four countries—Kenya (110), Rwanda (101), Uganda (363) and Tanzania (101)—which consisted of interviews using a structured questionnaire and a farm site visit. The gender of the survey respondent was noted on all questionnaires and a greater proportion of survey respondents in all countries were female—61.3% of all respondents (Fig. 3). The reported proportion of females involved in sweetpotato production in all countries was also significantly higher than that of males. The gender difference was highest in Uganda where the percentage of farmers involved in sweetpotato production who were female was 66% (P < 0.001). In Kenya, 54.5% (P = 0.035) of farmers involved in production were female; in Rwanda 56.4% (P = 0.015) and in Tanzania 55.4% (P = 0.043).

#### 3.1.2 Ranking of sweetpotato by gender

**Fig. 4:**
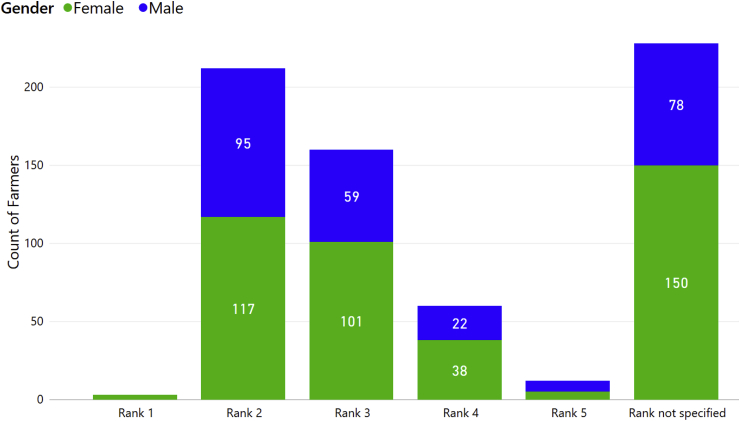
The results of the survey ranking the importance of sweetpotato as a staple crop, divided by the gender of the respondent and using a 1–5 scale where 1 is the most important and 5 the least important. Surveyed farmers were from Uganda (n = 363 surveys), Kenya (n = 110), Tanzania (n = 101) and Rwanda (n = 101).

We asked all survey participants to rank sweetpotato as their household's staple food using a 1–5 scale with 1 being the most important and 5 the least important. We assessed the proportion of men and women that ranked the crop either 1 or 2, and found no significant gender differences: 46% of women compared with 52% of men (P = 0.151) (Fig. 4). Overall, 48% ranked it as either 1 or 2, with almost all survey respondents ranking sweetpotato 2 (Fig. 4). We also found no significant differences in the proportion of women (89%) and men (84%) who ranked the crop 2 or 3 (P = 0.129).

#### 3.1.3 Ranking of sweetpotato by country

**Fig. 5:**
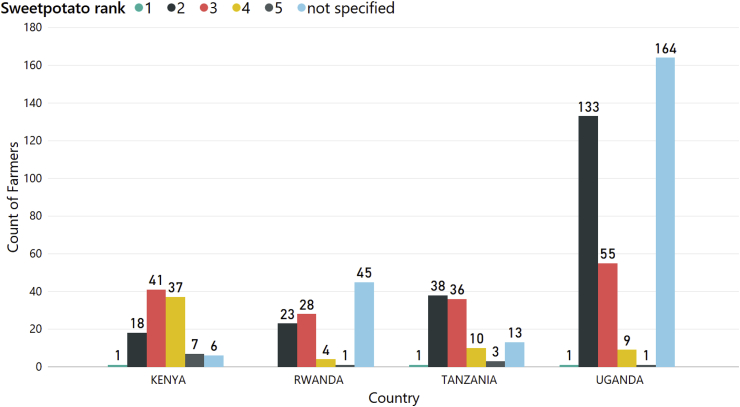
Ranking of sweetpotato as a staple crop by farmers in Uganda (n = 363 surveys), Kenya (n = 110), Tanzania (n = 101) and Rwanda (n = 101).

On average, 47% of the farmers ranked sweetpotato as their second staple food after cassava in all countries. However, there were differences in ranking between countries (P < 0.001). The proportion of the respondents who gave rank 1 or 2 by country varied: the crop was ranked lowest in Kenya (18.1%), was ranked mostly a 2 or 3 in Rwanda and Tanzania (41% and 44%, respectively) and mostly ranked a 2 in Uganda (67%) (Fig. 5). One-third of responses did not specify a rank.

#### 3.1.4 Sweetpotato production trends

Farmers were asked if they had observed trends in sweetpotato production over the past five years. This question was intended to provide an indication of perceived sweetpotato production levels per country. In Kenya, 4.5% of respondents reported a decrease in production and 56.4% reported an increase. The corresponding percentages were 51.5% (decrease) and 9.9% (increase) in Rwanda, 63.4% and 22.8% in Tanzania and 27.3% and 40.8% in Uganda (Table 1). Within each country, the differences in the number of respondents reporting a decrease or an increase were significant (P < 0.001); however, across the four countries, the difference between the respondents who reported decreases (33%) and increases (36%) was non-significant (P = 0.104). Across all countries, 17.8% of respondents reported that production had remained the same (Table 1).

**Table 1:** Trends in sweetpotato production over the past five years in Uganda (n = 363 surveys), Kenya (n = 110), Tanzania (n = 101) and Rwanda (n = 101), according to the knowledge of the farmers surveyed.

Country	Decreased (%)	Increased (%)	Unchanged (%)	*P*-value
Kenya	4.5	56.4	37.3	<0.001
Rwanda	51.5	9.9	38.6	<0.001
Tanzania	63.4	22.8	9.9	<0.001
Uganda	27.3	40.8	8.3	<0.001
All countries (N = 675)	32.6	36.0	17.8	<0.104

#### 3.1.5 Sweetpotato cultivated area per country

We took field measurements to estimate the area of each sweetpotato farm and then asked each farmer to tell us how much of their land was under cultivation by sweetpotato and other crops. Our estimates for total cultivated land area under sweetpotato was 386.4 ha and for all other crops 2069.4 ha. Across all four countries, the proportion of sweetpotato compared with other cultivated crops varied: 19.7% of cultivated land was used for sweetpotato in Kenya, 10.9% in Rwanda, 23.8% in Tanzania and 17.7% in Uganda (Table 2). These estimates are inherently biased as we only interviewed farmers growing sweetpotato; thus, these data can only be used to evaluate farms on which sweetpotato is grown.

**Table 2:** Reported area of fields, total land under cultivation and the proportions of fields used to grow sweetpotato and other crops according to farmers in Uganda (n = 363 surveys), Kenya (n = 110), Tanzania (n = 101) and Rwanda (n = 101).

Country	Total land under cultivation (ha)	Other crops (ha)	Sweet-potato area (ha)	Proportion sweetpotato relative to total cultivated area (%)	Mean field size (ha)	Min field size (ha)	Max field size (ha)
Kenya	247.57	198.85	48.72	19.7	2.26	0.01	4.0
Rwanda	202.19	180.06	22.13	10.9	2.00	0.01	2.5
Tanzania	296.25	225.86	70.39	23.8	3.00	0.13	3.0
Uganda	1598.88	1315.97	282.91	17.7	4.92	0.01	6.0
All countries (N = 675)	2344.89	1920.74	424.15	18.1	3.05	0.04	3.1

Across all four countries, sweetpotato is a subsistence crop grown on small areas of less than 0.4 ha on average. In Uganda, sweetpotato farm sizes were in the range of 0.01–6.00 ha; and 0.01–4.0 ha in Kenya, 0.13–3.0 ha in Tanzania and 0.01–2.5 ha in Rwanda (Table 2). On average, the proportion of sweetpotato cultivated areas ranged from 10.9 to 23.8% in all four countries (Table 2).

#### 3.1.6 Sweetpotato cultivated areas within each AEZ

**Fig. 6:**
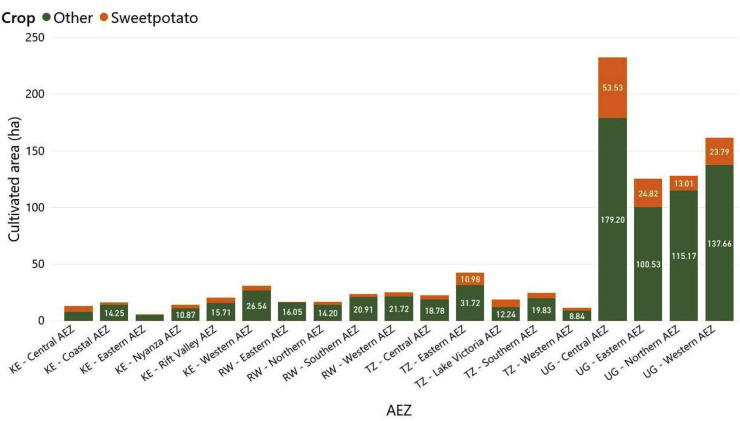
Sweetpotato cultivated areas compared with other crops across the different agroecological zones in Kenya (KE), Rwanda (RW), Tanzania (TZ) and Uganda (UG), according to farmers in Uganda (n = 363 surveys), Kenya (n = 110), Tanzania (n = 101) and Rwanda (n = 101).

The proportion of the sweetpotato-cultivated area within each AEZ per country varied. In Kenya, the proportion of sweetpotato cultivated relative to other crops was highest in the Central AEZ (40.1%), followed by Nyanza (23.8%), Rift Valley (22.3%), Western (13.7%), Coastal (12.2%) and Eastern (7.4%) (Fig. 6). In Rwanda, the highest proportion of sweetpotato cultivated was in Northern AEZ (14.94%), followed by Western (13.2%), Southern (11.2%) and Eastern AEZ (3.3%). In Tanzania, the highest proportion of sweetpotato was in the Lake Victoria AEZ (33.6%), followed by Eastern (25.5%), Western (22%), Southern (19.7%) and Central AEZ (17.5%). In Uganda, the highest proportion of sweetpotato was in Central AEZ (23%), followed by Eastern (19.4%), Western (14.7%) and Northern (10.14%) (Fig. 6).

#### 3.1.7 Seedbed type by country

**Fig. 7:**
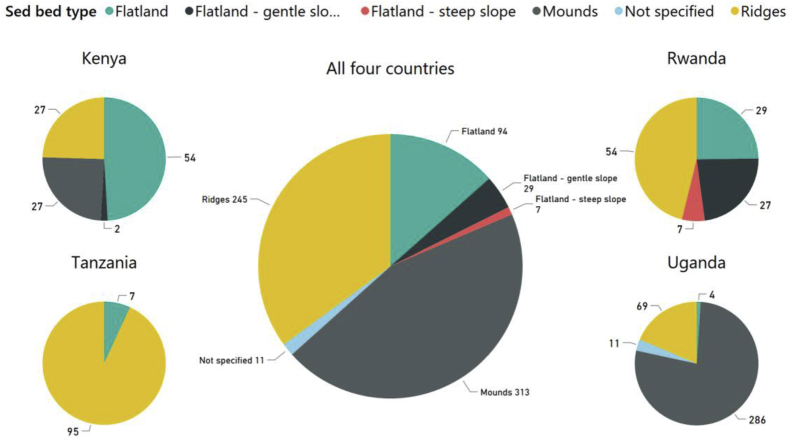
Sweetpotato seedbed types reported by farmers surveyed in Uganda (n = 363 surveys), Kenya (n = 110), Tanzania (n = 101) and Rwanda (n = 101), and in all four countries combined (N = 675).

Of the farms visited in the four countries, 43.3% had mounds as the seedbed type, followed by ridges (35%), flatland (13.5%) and mixed mounds and flatland (3.5%) (Fig. 7). Sweetpotato seedbed types varied greatly by country. In Tanzania, ridges were by far the most common type (93.1%), followed by flatland (5.9%) and mixed (1%) (Fig. 7). In Uganda, mounds were the most common (77.3%), followed by ridges (19.6%) and mixed (2.2%). In Rwanda, flatland was most common (63.0%), followed by ridges (37.0%). In Kenya, flatland was the most common (49.0%), followed by mounds (24.6%) and ridges (24.6%) (Fig. 7).

#### 3.1.8 Sweetpotato cropping systems

The field observations and surveys showed that across all four countries, 75.1% of farmers practiced pure cropping of sweetpotato and only 23.6% practiced intercropping (Table 3). These local practices did not vary much by country. In Uganda, 74.9% of farmers practiced pure cropping and 23.4% intercropping; in Kenya 73.6% (pure) and 24.6% (intercropping); in Rwanda 74.3% and 25.7%, and in Tanzania 78.2% and 20.8% (Table 3).

**Table 3:** Cropping systems reported by farmers surveyed in Uganda (n = 363 surveys), Kenya (n = 110), Tanzania (n = 101) and Rwanda (n = 101), and in all four countries combined (N = 675).

Country	Cropping system (%)		
	Pure cropping	Intercropping	Not specified
Kenya	73.6	24.6	1.8
Rwanda	74.3	25.7	0.0
Tanzania	78.2	20.8	1.0
Uganda	74.9	23.4	1.7
All countries (N = 675)	75.1	23.6	1.3

#### 3.1.9 Sources of planting material

**Fig. 8:**
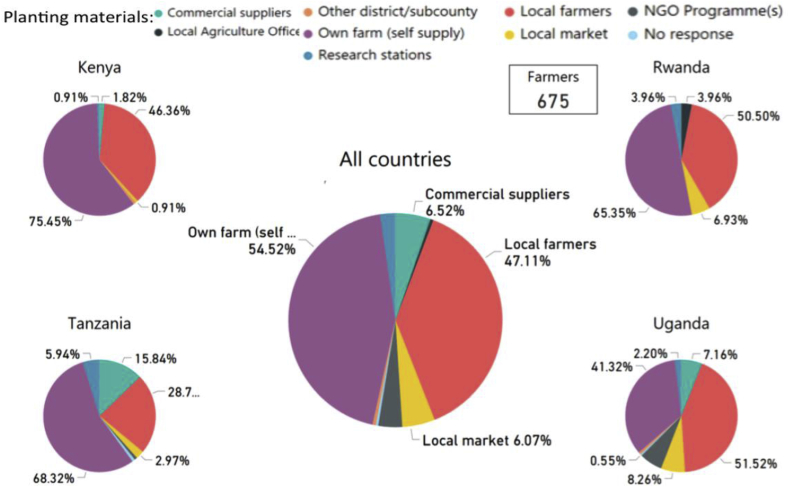
Source of planting materials reported by farmers surveyed in Uganda (n = 363 surveys), Kenya (n = 110), Tanzania (n = 101) and Rwanda (n = 101), and in all four countries combined (N = 675).

Across all countries, we found that the source of most planting material was from within a household's own farm (i.e. self-supply, 44.3%) and from other local farmers (38.3%). Overall, a small percentage of farmers sourced planting material from local markets (4.9%), commercial suppliers (5.9%), a non-governmental organization (NGO, 3.6%), a research station (2.3%), a local agricultural office (0.5%) or from other districts/sub-counties (0.5%) (Fig. 8). However, sources differed substantially by country. In Kenya, 74.5% of farmers sourced their planting materials from their own farms, followed by other farmers (46.4%), commercial suppliers (1.82%), research stations (0.91%) and local markets (0.91%) (Fig. 8). In Rwanda, 65.4% of farmers got planting materials from their own farms, followed by from other farmers at 50.5%, local market at 6.9%, NGO programs 4% and research stations at 4% (Fig. 8). In Tanzania, 68.3% of farmers got planting materials from their own farms, followed by other farmers (28.7%), commercial suppliers (15.8%), research stations (5.9%) and the local market (3%). In Uganda, a majority of farmers (51.5%) sourced their planting materials from other farmers (51.5%), followed by their own farms (41.3%), NGO programs (8.3%), local markets (8.3%), commercial suppliers (7.2%) and research stations (2.2%) (Fig. 8).

#### 3.1.10 Constraints to sweetpotato production

**Fig. 9:**
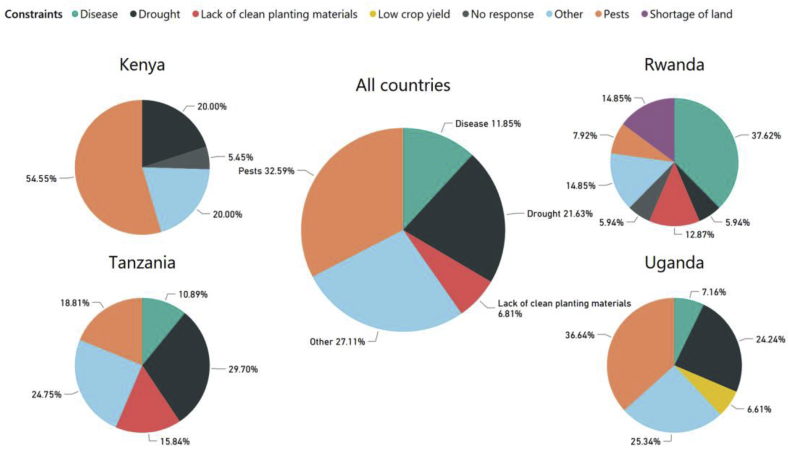
Most serious constraints to sweetpotato production reported by farmers surveyed in Uganda (n = 363 surveys), Kenya (n = 110), Tanzania (n = 101) and Rwanda (n = 101), and in all four countries combined (N = 675).

Farmers were asked to list and rank the constraints facing their sweetpotato production. Across all four countries, about 32.6% of farmers ranked pests as their number-one problem, followed by drought (21.6%), diseases (11.9%) and lack of disease-free planting materials (6.8%) (Fig. 9). Other notable constraints facing farmers were shortage of land, low crop yield, land exhaustion, poor soil quality and many other factors, which together contributed 27.1% (Fig. 9).

Farmers' constraints to sweetpotato production varied among the countries. In Kenya, the majority of farmers reported that pests (54.6%) were their main constraint followed by drought (20%), and to a lesser degree lack of disease-free planting materials, diseases, shortage of land, shortage of fertilizers, poor soils, animals and flooding (Fig. 9). In Rwanda, the largest proportion of farmers reported that disease (37.6%) was their number-one constraint, followed by shortage of land (14.9%), pests (7.9%), and drought (5.9%). Other constraints (14.9%) included shortage of fertilizers, poor soil quality, low crop yield, land exhaustion and low market price. In Tanzania, the largest proportion of farmers reported that drought (29.7%) was their major constraint, followed by pests (18.8%), lack of disease-free planting materials (15.8%) and diseases (10.9%). Other constraints included poor soil quality, no available market, labor shortage, low market price, shortage of land, shortage of fertilizers and weeds. In Uganda, the largest proportion of farmers reported that pests (36.6%) were the main constraint followed by drought (24.2%), diseases (7.2%) and low crop yield (6.6%) (Fig. 9). Other constraints included labor shortage, lack of disease-free planting materials, animals, land exhaustion, poor soil quality, shortage of land, weeds, flooding and rotting tubers.

### 3.2 Determination of local farmers' knowledge of sweetpotato diseases

**Fig. 10:**
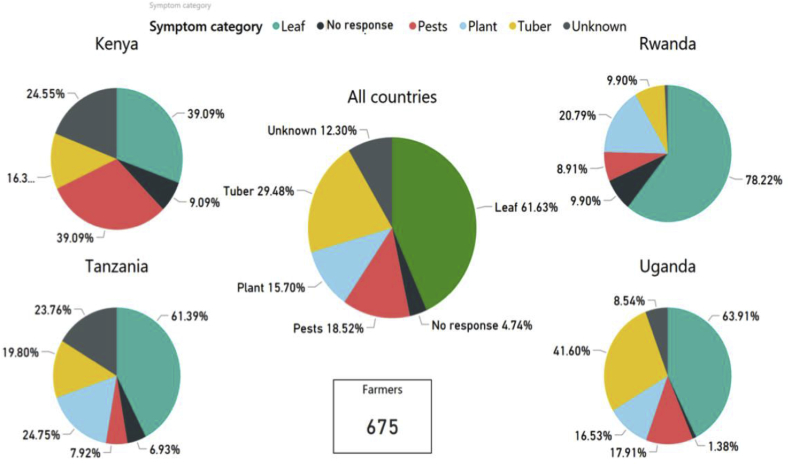
Sweetpotato disease symptoms observed on leaves, plants and tubers as reported by farmers surveyed in Uganda (n = 363 surveys), Kenya (n = 110), Tanzania (n = 101) and Rwanda (n = 101), and in all four countries combined (N = 675).

Farmers were asked to describe their knowledge of sweetpotato disease symptoms. Across all countries, most farmers (61.6%) positively identified disease symptoms on leaves); whereas fewer farmers associated disease with symptoms on root tubers (29.5%) or whole plants (15.7%), and only 18.5% recognized sweetpotato pests as being associated with sweetpotato disease (Fig. 10). Across the countries, correct identification of disease symptoms and signs varied. In Kenya, a large proportion of farmers associated leaf symptoms (39.1%) or presence of pests (39.1%) with disease, and few recognized disease symptoms on root tubers (16.3%). A large percentage of farmers (24.6%) did not recognize any of the disease signs or symptoms (Fig. 10). In Tanzania, 61.4% of farmers recognized disease symptoms on leaves, followed by whole plants (24.8%), and root tubers (19.8%), and by the presence of pests (7.9%). A large proportion of farmers (23.8%) in Tanzania did not recognize any of the signs or symptoms associated with diseases. In Rwanda, 78.2% of farmers associated symptoms on leaves with disease, followed by whole plants (20.8%), root tubers (9.9%) and pests (8.9%); 9.9% had no knowledge of disease signs and symptoms. In Uganda, a majority of farmers (63.9%) associated leaf symptoms with disease, followed by root tubers (41.6%), pests (17.9%) and whole plants (16.5%); 8.5% had no knowledge of disease signs and symptoms (Fig. 10).

#### 3.2.1 Farmers' knowledge on symptoms on sweetpotato plants

**Fig. 11:**
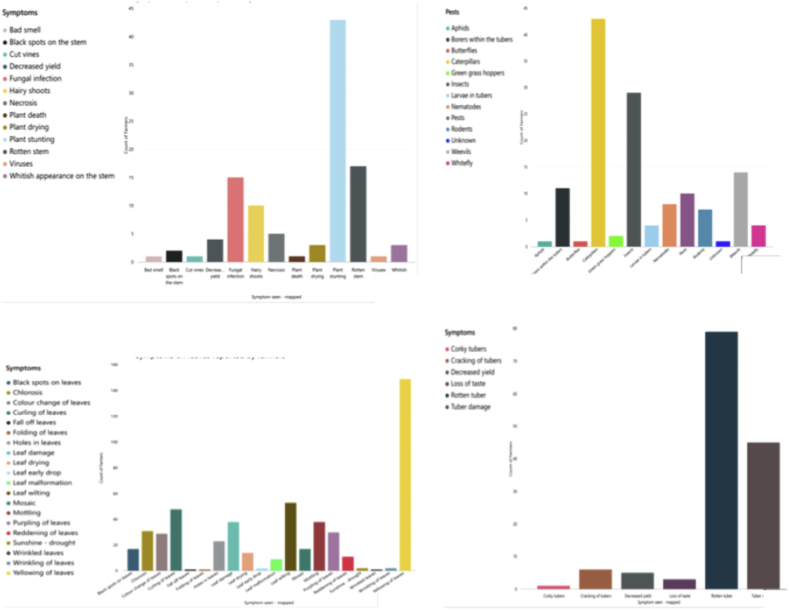
Observations associated with diseases and pests of sweetpotato plants, as reported by farmers surveyed in Uganda (n = 363 surveys), Kenya (n = 110), Tanzania (n = 101) and Rwanda (n = 101), and in all four countries combined (N = 675).

Farmers reported a variety of symptoms on leaves, including black spots, chlorosis, curling, holes, mosaic, purpling, reddening, wrinkling and yellowing as well as wilting, molting, drying, falling, folding and early dropping (Fig. 11). Leaf yellowing was most commonly reported (43.7%), followed by leaf wilting (15.6%) and leaf curling (12.7%) (Fig. 11). None of the farmers could distinguish whether these symptoms were related to viral, bacterial or fungal diseases. Furthermore, many farmers confused symptoms of diseases vectored by insects with the direct damage caused by insects feeding on the plants.

Farmers reported various disease symptoms on whole sweetpotato plants including bad smells, black spots on the stem, vines having cracks, signs of fungal infection, hairy shoots, necrosis, plant death, plant drying, plant stunting, signs of viruses, whitish appearance on stems and rotten stems; they also associated decreased yield with disease (Fig. 11). Plant stunting was reported by 43.9% of farmers, followed by rotten stems (17.3%) (Fig. 11).

Among the disease symptoms identified on root tubers, farmers reported corky texture, cracking, loss of taste, rotting and tuber damage as disease symptoms, without distinguishing or specifying the diseases (Fig. 11). Farmers also associated decreased yields with tuber-related disease.

#### 3.2.2 Pests as symptoms of sweetpotato diseases

Farmers associated pests with sweetpotato diseases. The major pests identified were aphids, borers, butterflies, caterpillars, green grasshoppers, nematodes, weevils and white flies (Fig. 11). Although these pests are vectors of pathogens causing diseases, the farmers confused symptoms of diseases vectored by these insects with the direct plant damage that resulted from insect feeding.

## 4 Discussion

In this work, we used a cross-country survey to assess local practices and knowledge of local production constraints and diseases to gain insight into sweetpotato production from the perspective of farmers. Our results provide strong evidence that pests, diseases, drought and lack of disease-free planting materials are the greatest constraints to sweetpotato production across all four countries. Although there are research organizations, government ministries of agriculture and NGOs in the four countries, sweetpotato pests and diseases [[36], [37], [38], [39], [40], [41]] remain major challenges in all the study areas.

Insects are known vectors for many plant viral and bacterial diseases [42], however, few studies have been undertaken in Rwanda, Uganda, Kenya and Tanzania to determine vector population dynamics and their capacity to transmit diseases. In the wider context of controlling these vectors, strategies should be developed and adapted for control of insects at the local scale. Farmers should also be trained to increase their awareness and knowledge of the common vectors and the diseases they transmit. Future efforts should be focused on improving farmers' abilities to identify and manage diseases, and on developing strategies suited for African settings, such as pesticides, low-cost pheromone-baited traps and breeding pest-resistant sweetpotato varieties. Farmers in these AEZ where diseases are common should be encouraged to use successful disease-management practices. For example, farmers should keep fields and surrounding areas free of weeds and volunteer plants, and ensure old crops are completely destroyed after harvest to remove sources of re-infection. Farmers can be encouraged to use certified disease-free planting material and to grow seedlings in greenhouses to limit the access of virus-vectoring insects.

Smallholder farmers find it challenging to identify the symptoms of fungal, bacterial and viral diseases; such symptoms can even be confusing for trained experts to differentiate. Recommended practices exist, but farmers in the four countries are either unaware of them or they are simply not being practiced in these regions. Farmers' field kits should be developed for extension staff and farmers to improve identification of these diseases. Our study showed that local farmers have limited knowledge of the practices that can be used to manage viral or bacterial diseases. Unless these farmers are trained, they will be unable to identify the symptoms and control these diseases. We recommend having plant clinics that would aid in the diagnosis of plant diseases and the training of farmers.

Although it was not part of the goal of this study to quantify disease symptoms in the surveyed farmers' fields, our field visits showed that a great proportion of fields had high levels of the symptoms associated with viral diseases. Viruses are widely considered to be of great economic importance in sweetpotato production [19,43], thus future studies should be undertaken to quantify the state of viral diseases in sweetpotato-growing AEZ. In addition, seed-vine multiplication companies should screen their materials for viral pathogens before they are distributed to farmers, and future studies should generate regional disease prevalence maps to guide seed-vine distribution for areas where disease pressure is high.

Our field survey data also showed higher involvement of females compared with males in the production of sweetpotato. Males and females ranked the crop similarly in importance. We speculate that both genders attribute importance to the crop because it provides the household with a staple food and provides food security. During our surveys, both females and males ranked the staple crop highly and the ranking did not significantly differ between the sexes. During the course of the surveys, we also observed more females than males tending their gardens, which may indicate the critical contribution of females in household food production. In Africa, women produce 70–75% of agricultural food [44,45]. In the East African region, and sub-Saharan Africa in general, sweetpotato is mainly cultivated by women and is referred to by some as a “female crop” [28]. Women in these regions can be hindered as a result of cultural differences, gender roles and responsibilities. Our study indicates that targeting women to disseminate appropriate sweetpotato technologies and extension services will lead to improved sweetpotato management and ultimately to increased sweetpotato production in households. Women should be empowered with hands-on skills in the management and control of pests and diseases, and they should be provided with disease-free planting materials.

Our results showed that sweetpotato fields in these four countries were generally grown as a single crop, with a moderate amount of intercropping practiced. Consistent with previous findings [46,47], the most common intercrops were cassava, banana, maize, beans and coffee. The ability of sweetpotato to quickly and vigorously produce dense foliage that rapidly covers the ground and outcompetes weeds is one reason why it is well suited to intercropping [48]. Intercropping seems to be practiced moderately among smallholder farmers because growing many crops in a single field can stabilize the household food supply throughout the year and improve land productivity. Bashaasha et al. [46] pointed out that farmers grew sweetpotato in mixtures due to shortage of vines, and the desire to extend harvesting periods, stabilize root yield and improve household food security. We believe that more farmers would integrate intercropping of sweetpotato with other crops if they were informed of the benefits of intercropping.

Despite farmers in Tanzania and Rwanda stating that there was reductions in sweetpotato production, the proportion of sweetpotato cultivated area is actually highest in Tanzania, followed by Kenya, Uganda and Rwanda [49] Sweetpotato continues to provide security in households and sweetpotato cultivation continues to expand in all four countries [50,51].

We found strong evidence that sweetpotato was grown as a subsistence crop by the majority of smallholder farmers on pieces of land of less than 0.4 ha. This can have consequences in sweetpotato production levels and food security in households because relatively small areas of land are being utilized to cultivate sweetpotato. Thus, these smallholder families risk having insufficient food if they do not produce other crops.

### 4.1 Conclusions and implications for food security

The findings of this study reaffirmed the importance of sweetpotato as a major source of food security to smallholder farmers in Rwanda, Uganda, Kenya and Tanzania. Sweetpotato was ranked by farmers as the second most important root crop after cassava in the region. With the current climate variability and changes, and the fast-growing population in Rwanda, Uganda, Kenya and Tanzania, sweetpotato remains a vital crop in overcoming food insecurity in these countries.

The failure of farmers to distinguish the symptoms of viral, bacterial and fungal diseases indicates there is a need to strengthen extension services to provide education to farmers so they can better manage these diseases. High involvement of women in sweetpotato production indicates that targeting women with knowledge and management strategies will be key to promoting increased yields at the household level and ensuring food security for families.

The high constraints of pests, diseases, drought and lack of disease-free planting materials reported by farmers in the four countries should be seriously considered. Lack of clean seed-vine multiplication programs in all four countries indicates the need to review government policies to address these constraints. Provision of disease-free planting materials and breeding for disease-resistant varieties should be a priority in all four countries.

## Figures and Tables

**Table:** Sweetpotato surveyed localities in Uganda, Kenya, Rwanda and Tanzania

Country	Year	Region	District	Farmer count	Average longitude	Average latitude
Kenya	2016	Central Kenya	Kirinyaga (Kerugoya/Kutus)	9	37.31333333	−0.603333333
Kenya	2016	Central Kenya	Murang'a	9	37.11777778	−0.843333333
Kenya	2016	Central Kenya	Nyeri	7	37.07285714	−0.455714286
Kenya	2016	Coastal Kenya	Kwale	4	39.435	−4.39
Kenya	2016	Coastal Kenya	Taita-Taveta (Wundanyi)	11	38.49818182	−3.725454545
Kenya	2016	Eastern Kenya	Embu	5	37.54	−0.536
Kenya	2016	Eastern Kenya	Machakos	4	37.325	−1.5225
Kenya	2016	Nyanza Kenya	Homa Bay	8	34.9575	−0.43375
Kenya	2016	Nyanza Kenya	Siaya	10	34.39	0.093
Kenya	2016	Rift Valley Kenya	Kericho	7	35.07428571	−0.398571429
Kenya	2016	Rift Valley Kenya	Nakuru	9	36.19888889	−0.068888889
Kenya	2016	Western Kenya	Bungoma	10	34.527	0.6
Kenya	2016	Western Kenya	Busia	7	34.16428571	0.317142857
Kenya	2016	Western Kenya	Kakamega	10	34.797	0.284
Rwanda	2015	Eastern Rwanda	Bugesera	7	30.16428571	−2.195714286
Rwanda	2015	Eastern Rwanda	Ngoma	10	30.551	−2.138
Rwanda	2015	Northern Rwanda	Gakenke	8	29.80875	−1.63625
Rwanda	2015	Northern Rwanda	Rulindo	8	29.91625	−1.73
Rwanda	2015	Southern Rwanda	Huye	10	29.714	−2.556
Rwanda	2015	Southern Rwanda	Kamonyi	6	29.88833333	−2.013333333
Rwanda	2015	Southern Rwanda	Muhanga	7	29.75428571	−2.105714286
Rwanda	2015	Southern Rwanda	Nyamagabe	10	29.51	−2.5
Rwanda	2015	Western Rwanda	Ngororero	9	29.61333333	−1.867777778
Rwanda	2015	Western Rwanda	Nyamasheke	8	29.035	−2.43
Rwanda	2015	Western Rwanda	Rubavu	8	29.3075	−1.69125
Rwanda	2015	Western Rwanda	Rusizi	10	29.032	−2.543
Tanzania	2016	Central Tanzania	Dodoma	10	35.944	−6.138
Tanzania	2016	Central Tanzania	Gairo	3	36.88666667	−6.143333333
Tanzania	2016	Central Tanzania	Ikungi	2	34.77	−5.355
Tanzania	2016	Central Tanzania	Manyoni	3	34.95666667	−5.573333333
Tanzania	2016	Central Tanzania	Singida	1	34.6	−5.57
Tanzania	2016	Eastern Tanzania	Bagamoyo	7	38.85714286	−6.544285714
Tanzania	2016	Eastern Tanzania	Gairo	11	36.83909091	−6.342727273
Tanzania	2016	Eastern Tanzania	Mkuranga	8	38.86375	−7.1225
Tanzania	2016	Lake Victoria Tanzania	Misungwi	8	33.0625	−2.8025
Tanzania	2016	Lake Victoria Tanzania	Mwanza	1	33.03	−2.74
Tanzania	2016	Lake Victoria Tanzania	Sengerema	9	32.73	−2.691111111
Tanzania	2016	Southern Tanzania	Madaba	9	35.67555556	−10.45222222
Tanzania	2016	Southern Tanzania	Mufindi	10	35.117	−8.416
Tanzania	2016	Southern Tanzania	Namtumbo	1	35.67	−10.47
Tanzania	2016	Southern Tanzania	Singida	1	34.6	−5.58
Tanzania	2016	Western Tanzania	Gairo	1	35.07	−8.39
Tanzania	2016	Western Tanzania	Kasulu	6	29.98166667	−4.84
Tanzania	2016	Western Tanzania	Kigoma	10	29.766	−4.808
Uganda	2015	Western Uganda	Bushenyi	10	30.163	−0.567
Uganda	2015	Western Uganda	Hoima	9	31.36444444	1.38
Uganda	2015	Western Uganda	Kabarole	10	30.205	0.598
Uganda	2015	Western Uganda	Kisoro	10	29.716	−1.296
Uganda	2015	Western Uganda	Masindi	10	31.728	1.705
Uganda	2015	Western Uganda	Mbarara	9	30.70777778	−0.516666667
Uganda	2016	Central Uganda	Luwero	10	32.497	0.879
Uganda	2016	Central Uganda	Masaka	10	31.846	−0.246
Uganda	2016	Central Uganda	Mpigi	10	32.047	0.096
Uganda	2016	Central Uganda	Mukono	11	32.75818182	0.477272727
Uganda	2016	Central Uganda	Soroti	1	33.64	1.71
Uganda	2016	Eastern Uganda	Iganga	10	33.568	0.54967
Uganda	2016	Eastern Uganda	Kamuli	11	33.25454545	0.948181818
Uganda	2016	Eastern Uganda	Kumi	10	33.907	1.543
Uganda	2016	Eastern Uganda	Mbale	8	34.23625	1.015
Uganda	2016	Eastern Uganda	Soroti	9	33.67777778	1.73
Uganda	2016	Northern Uganda	Dokolo	11	33.13	1.923636364
Uganda	2016	Northern Uganda	Gulu	13	32.32769231	2.804615385
Uganda	2016	Northern Uganda	Kitgum	13	32.9	3.259230769
Uganda	2016	Northern Uganda	Kole	11	32.77636364	2.186363636
Uganda	2016	Northern Uganda	Lamwo	12	32.75166667	3.449166667
Uganda	2016	Northern Uganda	Oyam	10	32.443	2.446
Uganda	2016	Western Uganda	Hoima	1	31.43	1.44
Uganda	2016	Western Uganda	Kabarole	1	30.23	0.7
Uganda	2016	Western Uganda	Mbarara	3	30.71	−0.52
Uganda	2017	Central Uganda	Masaka	10	31.752	−0.352
Uganda	2017	Central Uganda	Mpigi	10	32.315	0.263
Uganda	2017	Central Uganda	Mukono	10	32.838	0.348
Uganda	2017	Eastern Uganda	Iganga	10	33.471	0.63
Uganda	2017	Eastern Uganda	Mbale	10	34.225	1.094
Uganda	2017	Eastern Uganda	Soroti	10	33.653	1.729
Uganda	2017	Northern Uganda	Arua	10	31.123	3.284
Uganda	2017	Northern Uganda	Gulu	10	32.307	2.797
Uganda	2017	Northern Uganda	Kitgum	10	32.857	3.318
Uganda	2017	Northern Uganda	Lira	10	32.798	2.275
Uganda	2017	Northern Uganda	Moyo	10	31.748	3.647
Uganda	2017	Western Uganda	Bushenyi	10	30.238	−0.515
Uganda	2017	Western Uganda	Kabarole	10	30.304	0.724
Uganda	2017	Western Uganda	Masindi	10	31.726	1.725
				675		
